# Nerve transfers which have worked for me

**DOI:** 10.1186/1753-6561-9-S3-A19

**Published:** 2015-05-19

**Authors:** Anil Bhatia

**Affiliations:** 1 Joshi Hospital, Pune, Maharashtra 411004, India

## 

Distal nerve transfers have contributed enormously to the strategies in brachial plexus reconstruction. They help to overcome the pitfalls of nerve grafting from proximal root (or peripheral nerve) stumps and work, effectively, as tendon transfers. Although use of the intercostals for transfer to the musculocutaneous nerve was first described, using a graft of the ulnar nerve, in the 1960s, transfer of the spinal accessory to the suprascapular nerve was the first direct transfer for restoration of rotator cuff function. This has proved effective for stabilization of the paralysed shoulder in >80% of cases. It is not logical to expect a tiny muscle like the supraspinatus to lift the entire upper limb. In the presence of other functioning muscles such as the pectoralis major, latissimus dorsi and teres major, abduction up to 90 degrees is consistently achieved (i.e. in C56 brachial plexus injuries). Similarly, external rotation, too, is seen in the majority of cases.

In more extensive palsies (C5678 and C5T1 injuries), it is rare to achieve abduction > 45 degrees by this transfer alone. External rotation is seldom seen in such patients and a derotation osteotomy is necessary later on.

In these circumstances, transfer of the third intercostal to the pectoral nerve has been very useful to improve the stability of the shoulder and has enabled the patient to bring the arm in front of the trunk. In addition, the strength of the thoraco-brachial grasp is very useful.

Direct transfer of the intercostals to the musculocutaneous has stood the test of time. In my experience (>300 cases of this transfer), biceps stronger than grade 3 is achieved in 72% of cases. Initially, the patient has to strain and hold his breath. However, dissociation from respiration is achieved in most cases by 18 months.

I prefer to use three intercostals (4^th^, 5^th^ and 6^th^). In addition, as mentioned above, the third intercostal helps for innervation of the pectoralis major.

Although the spinal accessory to musculocutaneous nerve transfer is much simpler and has produced equally consistent restoration of biceps, there are several pitfalls. The long nerve graft implies a corresponding delay in appearance of function (usually 12-14 months). In addition, the phrenic nerve to suprascapular nerve transfer is, then, the only alternative for shoulder function. This is a very strong transfer but the function achieved is more difficult to control. Often, the patient is disturbed by involuntary abduction while coughing and sneezing.

In partial palsies, use of fascicles from the ulnar nerve for the biceps can be considered the benchmark. Oberlin’s description of this technique has changed our perception of the prognosis in C56 and C567 palsies. There have been innumerable reports of consistent results across the world and my own statistics bear out this optimism (87% in > 200 patients). Combination of a fascicle from the median nerve to the nerve to brachialis has apparently added to this consistency. In cases of infraclavicular lesions, I opt for nerve transfers for the musculocutaneous nerve in patients older than 45 years (in whom nerve grafting has proved less reliable) and in cases where the defect in the musculocutaneous nerve is extensive.

The need for re-innervation of the triceps has always been a matter of debate. The decision to operate extensive palsies is almost always taken early i.e. around 2 months from the accident. At that stage, the triceps and pectoralis major might appear completely paralysed. Transferring intercostals to the radial nerve branches to the triceps has resulted in contraction of the muscle. However, the patient does not see this and it is very difficult to instruct him for strengthening.

Eventually, there is a significant spontaneous restoration of the triceps along the branches that are not divided for the nerve transfer. I have observed this in several cases and am, now, reluctant to use the intercostals to triceps transfer in the primary operation. However, in patients presenting late (> 6 months) with persistent deficits, we should include these transfers to the triceps and pectoralis major. In this context, three intercostals have served better than just two.

There have been several reports of distal nerve transfers for flexion and extension of the fingers. In C567 palsies, the patient uses the intact finger extensors for extension of the wrist. Inevitably, the wrist drops when he/she makes a fist. In such cases, I have had to perform a tendon transfer (usually FDS of the middle finger) after restoration of elbow flexion and shoulder stability. However, Bertelli’s idea of transferring the pronator quadratus branch of the anterior interosseous nerve for the radial branch to the extensor carpi radialis brevis during the primary operation is a very good idea. I have used it > 10 times. I have noted consistent restoration of wrist dorsiflexion at the same time as the biceps.

Finally, cases of isolated involvement of the C8T1 roots are uncommon (2-4% of all brachial plexus injuries). Traditionally, the intact brachioradialis and extensor carpi radialis longus are used to restore flexion of the thumb and of the fingers respectively. However, opening the fist depends on the tenodesis effect of wrist palmarflexion using the flexor carpi radialis. Again, Bertelli’s ingenious use of the supinator branch of the radial nerve serves very well to restore extension of the fingers and thumb. The tendon transfers and the nerve transfer are performed together.

